# Braın abscess due to *Streptococcus intermedius* secondary to mastoiditis in a child

**DOI:** 10.1186/s40064-015-1608-0

**Published:** 2015-12-23

**Authors:** Nurhayat Yakut, Eda Kepenekli Kadayifci, Ayse Karaaslan, Serkan Atici, Gulsen Akkoc, Sevliya Ocal Demir, Adnan Dagcinar, Fatih Akbulut, Ahmet Soysal, Mustafa Bakır

**Affiliations:** Division of Pediatric Infectious Diseases, Department of Pediatrics, Marmara University School of Medicine, Istanbul, Turkey; No 41, Fevzi Cakmak mahallesi, Ustkaynarca, Pendik, Istanbul, Turkey; Department of Neurosurgery, Marmara University School of Medicine, Istanbul, Turkey

**Keywords:** Brain abscess, *Streptococcus intermedius*, Child, Mastoiditis

## Abstract

**Background:**

Brain abscess is a rare but serious, life-threatening infection in children. It may arise from parameningeal infections such as otitis media, sinusitis and mastoiditis.

**Case description:**

A ten-year-old boy with the diagnosis of glycogen-storage disease and obesity was admitted to the emergency room with complaints of vomiting, decreased level of consciousness, imbalance on walking. On neurological examination, the patient was ataxic. His cranial magnetic resonance imaging (MRI) examination showed mastoiditis on the right side and 39 × 34 mm abscess formation with surrounding edema on the right cerebellar hemisphere. The patient underwent surgery to drain the abscess, microbiological samples were obtained and empirical antibiotic treatment with vancomycin and piperacillin–tazobactam were started. Postoperative cranial MRI examination showed that the lesion regressed 10 × 10 mm with a reduction in the edema. On the second week of the treatment, the antibiotics were switched to vancomycin and meropenem because of the relapsing fever. The therapy was continued for 6 weeks. A final MRI (after completing antibiotherapy) showed resolution of the cerebellar abscess. The child’s clinical condition improved and he was discharged without any sequelae.

**Discussion and evaluation:**

Children with congenital heart disease and an immonocompromised state are particularly at risk. *Streptococcus intermedius* is usually a commensal microorganism in the normal flora of the mouth which can cause brain abscess rarely in children. Brain abscess induced mortality rates are still relatively high, even with the advancement of imaging technologies, the combination of surgical drainage and antimicrobial therapy.

**Conclusion:**

This case is one of the few reported cases of cerebellar abscess caused by *S. intermedius* in an immunocompetent child, due to its low virulence, a rare occurence and timely management resulting in fully healed.

## Background

Brain abscess is a rare but serious and life-threatening infection during childhood (Hanche-Olsen et al. 2012). The most frequent age group in children is 4–7-year olds (Ftaziet et al. 2008). It may arise from parameningeal infections such as otitis media, sinusitis and mastoiditis. Children with congenital heart disease and an immonocompromised state are particularly at risk (Kao et al. 2008). The clinical presentation of a brain abscess can vary depending on the location, the child’s age, the host immune status and may be subtle (Goodkin and Pomeroy 2003). The most common symptoms are fever, headache and focal neurological dysfunction occur in only 9–28 % of the pediatric cases (Yogev and Bar-Meir 2004). The causative pathogens in children are aerobic and anaerobic streptococci (60–70 % of cases), gram-negative anaerobic bacilli (20–40 %) followed by Enterobacteriaceae (20–30 %), *S. aureus* (10–15 %) and fungi make up less than 5 % (Arias and Murray 2010). *Streptococcus intermedius*, a member of the *Streptococcus milleri* group, is usually a commensal microorganism in the normal flora of the mouth and upper airways. Although viridans streptococci are considered low virulence microorganisms in immunocompetent individuals, they can cause invasive pyogenic infections such as brain abscesses and bone infections (Doern and Burnham 2010). We report a brain abscess due to *S. intermedius* in a child with the diagnosis of glycogen-storage disease.

## Case description

**Fig. 1:**
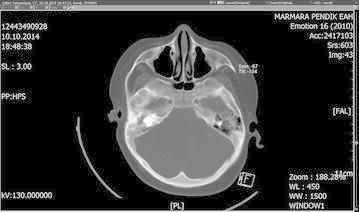
Mastoiditis on the right side

**Fig. 2:**
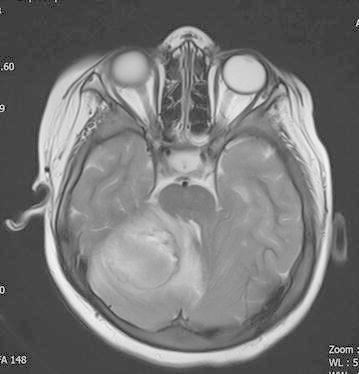
Preoperative cranial MRI showed a 39 × 34 mm mass lesion

**Fig. 3:**
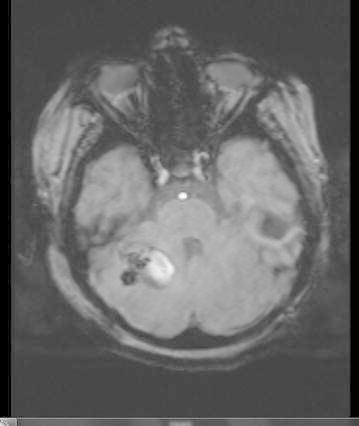
Postoperative cranial MRI showed a regressed lesion

A ten-year-old boy with the diagnosis of glycogen-storage disease and obesity was admitted to the emergency room with complaints of vomiting, decreased level of consciousness, imbalance on walking. Additionally there was history of ear pain and discharge for the last several days. On neurological examination, the patient was ataxic. He had no fever or neck stiffness on physical examination. Laboratory tests showed a white blood cell of 10,600/µl (normal range 4000–10,000), C-reactive protein of 10.2 mg/dl (normal range 0–5), erythrocyte sedimentation rate of 80 mm/h (<20 mm/h) and normal biochemistry values. The peripheral blood cultures were obtained before the antimicrobial treatment and incubated at Bact Alert (Biomerièux, France) device for both aerobe and anaerobic cultures and they remained sterile. His cranial magnetic resonance imaging (MRI) examination showed mastoiditis on the right side (Fig. 1) and 39 × 34 mm abscess formation with surrounding edema on the right cerebellar hemisphere (Fig. 2). Echocardiography of the patient was performed and any congenital or acquired heart disease was not detected. The patient underwent surgery to drain by puncture of the cystic portion of the abscess, microbiological samples were obtained and empirical antibiotic treatment with vancomycin and piperacillin–tazobactam were started. On gram-stain gram-positive cocci were seen. One day later Vitek MS identified the microorganism as *S. intermedius* and reliability was 99.9 %. The e-test (Biomerièux, France) used to describe antimicrobiological susceptibility showed that *S. intermedius* which was susceptible to penicilin, ampicillin, ceftriaxone and cefuroxime. Postoperative cranial MRI examination showed that the lesion regressed 10 × 10 mm with a reduction in the edema (Fig. 3). On the second week of the treatment, the antibiotics were switched to vancomycin and meropenem because of the relapsing fever and possible healthcare associated infections. The therapy was continued for 6 weeks. A final MRI (after completing antibiotherapy) showed resolution of the cerebellar abscess. The child’s clinical condition improved and he was discharged without any sequelae.

## Discussion and evaluation

Brain abscesses are purulent collections in the parenchyma of the central nervous system or meningeal spaces. The most common origin of brain abscess in children is direct or indirect (blood-borne invasion) cranial infection arising from the middle ear, paranasal sinuses or teeth. Ear and mastoid infections are associated with formation of an abscess at the temporal or cerebellar locations. Contiguous spread of mastoiditis and the other contiguous site infections may result as brain abscess. We thought that the route of infection in our patient is the direct invasion of bacteria from mastoid antrum to cerebellum. Hematogenous spread was also possible, however the blood cultures remained sterile. We could collect only a small blood sample for the peripheral vein culture, this may be the reason the cultures remained sterile. The high incidence of sterile cultures reported in the literature is probably because of the inadequacy of bacteriological procedures. Although relatively rare in children and the mortality rate seems to be decreasing, they still have a high mortality and morbidity (Frazier et al. 2008; Shachor-Meyouhas et al. 2010). Immunocompromised state, congenital cyanotic heart disease, head trauma and neurosurgical procedures can ease abscess formation. The most common preceding infections are face and head infections such as sinusitis, otitis and meningitis (Yogev 2008). In a multicenter clinical study, sinusitis is reported as the commonest preceding infection of brain abscess in children (Felsenstein et al. 2013). Predisposing factors for viridans streptococcal brain abscess include chronic otitis media, congenital heart disease, head trauma (Su et al. 2001). Predisposing condition in our case is mastoiditis but without chronic otitis media. It can occur in different region of the central nervous system such as parenchyma and subdural space. Temporal lobe is the most common site. Cerebellar abscesses are rare compared to the other locations of the central nervous system. The symptoms can vary depending on the location of abscess. Therefore the clinicians should be aware of children presenting with ear and cranial symptoms concomitantly. After the diagnosis of brain abscess has been established, a careful search should be made for a source of infection serving as a site of origin for hematogenous spread or direct inoculation of organisms into the central nervous system. In addition to obtaining data from the history and physical examination, the physician should extend the MRI or CT evaluation to include the mastoid antrum and paranasal sinuses. An echocardiogram should be performed to assess for underlying heart disease. Other testing should be performed by the history and physical examination findings. Although *S. intermedius* is a commensal microorganism in the normal flora of the mouth and upper airways, it can become a pathogen of brain abscess in children (Petti et al. 2008). This organism is usually susceptible to many antibiotics used to treat central nervous system bacterial infections. In our patient, antibiotic susceptibilities were determined by penicillin e-test (Biomerièux, France) and disk diffusion on Mueller–Hinton agar supplemented with 5 % defibrinated horse blood. Minimal inhibitory concentrations (MICs) for penicillin were 0.06 μg/ml (<0.12 sensitive) and vancomycin 23 μg/ml (>17 sensitive), respectively, by e-test in accordance Clinical and Laboratory Standards Institute guideline. It can be successfully treated by the combination of surgical approach and appropriate antibiotics. Only a few similar cases have been already reported in literature. Choudhury et al. reported a case of a 52-year-old man who had undergone orthotopic liver transplantation for end-stage liver disease and who developed a cerebellar abscess caused by Listeria monocytogenes (Choudhury et al. 2013). Hischebeth et al. reported a case of a 25-year-old man with drug abuse with a cerebellar abscess due to *Fusobacterium nucleatum* (2014). A brief report of 83 adult patients with brain abscess showed that 32 patients had mastoiditis and there is no case with cerebellar abscess (Faraji-Rad and Samini 2007). Garayev et al. reported a 6-year-old girl with cerebellar abscess secondary to bone destruction during mastoidectomy (2007). There are no guidelines for the management of brain abscess in children so approach of pediatric brain abscess is variable. Optimal therapy is a combination of surgical drainage and antibiotic treatment with isolated medical treatment in selected cases such as neurologically intact children with small abscess. There are two surgical approaches to brain abscess. First is the total excision of the lesion with microsurgical technique. The other one is performing drainage by puncture of the cystic portion of the abscess. Puncture technique has been preferred for last 20 years as a safer, easier and highly effective drainage method. By this method mass effect of the abscess is eradicated and sufficient material for microbiological investigation is obtained. Patient’s clinical findings of imbalance and decreased level of consciousness were related to the location of the lesion which was in the right cerebellar hemisphere. While imbalance was result of the cerebellar dysfunction, decreased level of consciousness showed brain stem compression. Because of these findings, our patient underwent operation for urgent decompression. Such a lesion located in the posterior fossa with a close relationship with petrous and mastoid bones usually accepted as a brain abscess. Surrounding hyperintense rim with GD uptake, cystic appearance of the lesion, clinical symptoms of infectious nature strongly suggest this pathology. Low or high grade gliomas, hemangioblastomas, metastatic tumors (which are very rare in children), acute demyelinating disease may all show the similar radiological features but when the patient was investigated and examined carefully the brain abscess should be considered firstly in differential diagnosis. The choice and duration of antibiotics is still unclear (De Louvois et al. 2000). Brain abscess induced mortality rates are still relatively high, even with the advancement of imaging technologies, the combination of surgical drainage and antimicrobial therapy (Goodkin et al. 2004).

## Conclusion

This case is one of the few reported cases of cerebellar abscess caused by *S. intermedius* in an immunocompetent child, due to its low virulence, a rare occurence and timely management resulting in fully healed.
